# Development of a microbial dewaxing agent using three spore forming bacteria

**DOI:** 10.1186/s40643-024-00795-z

**Published:** 2024-08-08

**Authors:** Xiaoyan Guo, Xutao Zhao, Lizhu Li, Haibo Jin, Jianjun Wang

**Affiliations:** 1 College of New Materials and Chemical Engineering, Beijing Institute of Petrochemical Technology, Beijing, People’s Republic of China; 2 Beijing Key Laboratory of Fuels Cleaning and Advanced Catalytic Emission Reduction Technology, Beijing, People’s Republic of China; 3 CAS Key Laboratory of Microbial Physiological and Metabolic Engineering, Institute of Microbiology, Chinese Academy of Sciences, Beijing, 100101 P.R. China

**Keywords:** Alkane monooxygenase, Microbial enhanced oil recovery, Microbial wax removal agent, Surfactin

## Abstract

Microbial enhanced oil recovery (MEOR) is a cost effective and efficient method for recovering residual oil. However, the presence of wax (paraffin) in residual oil can substantially reduce the efficiency of MEOR. Therefore, microbial dewaxing is a critical process in MEOR. In this study, a bacterial dewaxing agent of three spore-forming bacteria was developed. Among these bacteria, *Bacillus subtilis* GZ6 produced the biosurfactant surfactin. Replacing the promoter of the surfactin synthase gene cluster (*srfA*), increased the titer of surfactin in this strain from 0.33 g/L to 2.32 g/L. The genetically modified strain produced oil spreading rings with diameters increasing from 3.5 ± 0.1 to 4.1 ± 0.2 cm. The LadA F10L/N133R mutant was created by engineering an alkane monooxygenase (LadA) using site-directed mutagenesis in the *Escherichia coli* host. Compared to the wild-type enzyme, the resulting mutant exhibited an 11.7-fold increase in catalytic efficiency toward the substrate octadecane. When the mutant (*pIMPpladA2mu*) was expressed in *Geobacillus stearothermophilus* GZ178 cells, it exhibited a 2.0-fold increase in octadecane-degrading activity. Cultures of the two modified strains (*B. subtilis* GZ6 (*pg3srfA*) and *G. stearothermophilus* GZ178 (*pIMPpladA2mu*)) were mixed with the culture of *Geobacillus thermodenitrificans* GZ156 at a ratio of 5:80:15. The resulting composition increased the rate of wax removal by 35% compared to the composition composed of three native strains. This study successfully developed a multi-strain bacterial agent with enhanced oil wax removal capabilities by genetically engineering two bacterial strains.

**Supplementary Information:**

The online version contains supplementary material available at 10.1186/s40643-024-00795-z.

## Background

Non-renewable fossil fuels currently account for approximately 80–90% of the global energy supply (Norouzi et al. 2020). According to a report by the Organization of Petroleum Exporting Countries, by the end of 2023, the global total oil reserves reached 1.56 trillion barrels. Modern oil production processes can recover around 65% of crude oil by using primary and secondary extraction methods (Shibulal et al. 2014). However, approximately 35% of the original oil remains as residual oil (RO) in depleted oil fields, which is difficult to recover because of its high viscosity and low fluidity (Hall et al. 2003). To recover RO, various techniques have been developed over the years, which can be classified into enhanced oil recovery and tertiary recovery methods. Currently, three main methods of enhanced oil recovery are used in practical oilfield applications: chemical flooding, thermal enhanced oil recovery, and microbial methods (Shibulal et al. 2014). Although the first two methods achieve high recovery efficiencies, they also have drawbacks (Murungi and Sulaimon 2022). For example, the addition of chemicals often leads to secondary pollution, and the application of heat over a large oilfield area substantially increases costs (Zhao et al. 2023).

To overcome these limitations, scientists have developed a non-toxic and eco-friendly technique for oil recovery using microbes. This method is known as microbial enhanced oil recovery (MEOR) and it is particularly noteworthy because of its low operating costs and high recovery rates (Shibulal et al. 2014). MEOR utilizes the hydrocarbon metabolism of microbes to alter the physical properties and composition of RO, thereby rendering RO more readily recoverable (Astuti et al. 2019). The primary target of the MEOR process is the heavy oil fraction, which constitutes approximately 15% of total RO (Zhang et al. 2020). Heavy oil is a complex mixture composed mainly of alkanes (paraffins), cycloalkanes (naphthenes), and aromatic hydrocarbons. The majority of heavy oil components are saturated alkanes, of which those with carbon chain lengths exceeding C18 are classified as waxes. At room temperature, this fraction of saturated alkane is present in the solid state (Zhang et al. 2020). During the extraction of crude oil from high-temperature underground oil wells, the decreasing temperature results in the precipitation of waxes. The result is the formation of wax crystals that adhere to oil wells and oil production equipment, thus considerably reducing oil recovery efficiency. The MEOR process mainly deals with waxes and is, therefore, actually a dewaxing process. The microorganisms involved in MEOR also act as dewaxing agents. Waxy crude oil is a common feedstock problem encountered by the petroleum industry. Statistical data show that waxy crude oils account for up to 90% of the total crude oil produced from Chinese oil fields. Most of these crude oils have wax contents above 20%, with certain samples containing up to 40% wax (Wang et al. 2013). Currently, microbial dewaxing technologies are used in about 65% of all oil fields in China (Bian et al. 2021).

The microbial dewaxing process enhances heavy oil recovery through two mechanisms: increasing the solubility of hydrophobic compounds and degrading complex compositions (Wu et al. 2022). Bacteria are the primary microorganisms used for microbial dewaxing and during the process, they produce several bioactive molecules, the most important of which are biosurfactants. These biosurfactants prevent wax crystals from aggregating by creating polar surfaces that are not conducive to crystal growth, thus keeping the wax in a highly dispersed state, and enabling its easy transport by oil flow (Wang et al. 2022). In addition, dewaxing bacteria are capable of degrading waxes, such as heavy hydrocarbons with 22 to 40 carbon atoms, as demonstrated by *Pseudomonas* species (Etoumi et al. 2008). Chen et al. (2017) reported that a bacterium belonging to the *Dietzia* genus can metabolize n-alkanes ranging from C14 to C31 and achieve high degradation rates at neutral and slightly alkaline pH levels. In conclusion, the production of biosurfactants and the degradation of hydrocarbons are crucial capabilities of dewaxing strains. To optimize this biotechnology, it is essential to isolate native microbes with superior capabilities in both aspects. Alternatively, microbial chassis cells can be modified through gene editing to enhance the yield of biosurfactants, or protein engineering can be employed to enhance the catalytic efficiency of relevant metabolic enzymes within hydrocarbon metabolic pathways.

This paper reports the development of a tri-strain microbial dewaxing agent consisting of a genomically modified *Bacillus subtilis* strain, an alkane monooxygenase-enhanced *Geobacillus stearothermophilus* strain, and a native *Geobacillus thermodenitrificans* strain. The dewaxing rate for this tri-strain compound was significantly higher than the formulation containing the native forms of these three strains. According to references, the vast majority of microbial dewaxing agents that have been developed so far are native microbial strains (Patel et al. 2015). In this study, genetic editing and protein engineering methods were employed to modify dewaxing strains. The performances of the single modified strains and the combined agent were greatly enhanced, indicating that these two approaches are effective tools in the development of microbial dewaxing agents. The agent developed is currently being field tested in several Chinese oil wells.

## Methods

### Chemicals, bacterial strains, plasmids, and culture media

Flavin mononucleotide (FMN), octadecane and surfactin were purchased from Acros (Beijing, China). Crude oil samples (alkane content, 19.4% ) were provided by the PetroChina Liaohe Petrochemical Refinery. Other reagents used were analytical grade and commercially available. *Escherichia coli* ) cultures were grown in Lysogeny Broth (LB) medium at 37 °C. *E. coli* TOP10 cells (TransGen Biotech, Beijing, China) were used for plasmid construction. The pET30a plasmid (Invitrogen, California, USA) was used for routine cloning and expression in *E. coli* BL21(DE3). Bacterial clones carrying the desired recombinant plasmids were selected by supplementing growth media with specific antibiotics.

The enrichment medium consisted of 0.2% crude oil, NaNO_3_ 0.2%, (NH_4_)_2_SO_4_ 0.1%, NaCl 0.5%, MgSO_4_ 0.025%, KH_2_PO_4_ 0.5%, K_2_HPO_4_ 1%.

Medium 1(for bio-surfactant producing bacteria): sucrose 0.5%, NaNO_3_ 1%, K_2_HPO_4_ 0.3%, CaCl_2_ 0.012%, MgSO_4_ 0.024%, FeSO_4_ 0.012%, Na_2_MoO_4_ 0.008%, yeast extract 0.05%.

Medium 2 (for crude oil degrading bacteria): sucrose 0.5%, NaCl 0.3%, (NH_4_)_2_SO_4_ 0.15%, MgSO_4_ 0.02%, NaNO_3_ 0.3%, KH_2_ PO_4_ 0.01%, K_2_HPO_4_ 0.05%, FeSO_4_ 0.001%, yeast extract 0.05%.

Medium C18 (for *pIMPpLad2Mu* expression in *Geobacillus stearothermophilus*): octadecane 0.3%, NaCl 0.5%, (NH_4_)_2_SO_4_ 0.15%, MgSO_4_ 4.1%, KH_2_ PO_4_ 4.0%, K_2_HPO_4_ 4.1%, (NH_4_)SO_4_ 4.1%.

### Soil sample collection

Crude oil contaminated soil samples were collected from the Huaziping well at the Ansai Oilfield (Latitude 34°35’48"N, Longitude 108°95’35"E) located in North Shaanxi, Shaanxi Province, China. These samples were collected from a layer approximately 10 cm below the soil surface and placed into sterilized bags. Debris was removed from samples prior to screening experiments.

### Bio-surfactant producing bacteria screening

A total of 50 mL of enrichment medium was inoculated with 10 g of crude oil-contaminated soil and cultured on a rotary shaker at 40 °C and 220 RPM for 48 h. After enrichment, cultures were diluted 10-fold with sterile water. Next, 0.1 mL aliquots were plated on Medium 1 plates and incubated for 48 h at 40 °C. Subsequently, individual colonies from these plates were inoculated into 5 mL of Medium 1 and grown on a rotary shaker at 40 °C and 220 RPM for 48 h. Finally, the culture supernatant was collected and used for oil spreading assays. Oil spreading assays were conducted following the method as described by Morikawa et al. (2000). Each experiment was replicated three times.

### Screening crude oil degrading bacteria

A total of 50 mL of enrichment medium was inoculated with 10 g of crude oil-contaminated soil and cultured on a rotary shaker at 60 °C and 220 RPM for 48 h. After enrichment, cultures were diluted 10-fold with sterile water, and 0.1 mL of the diluted culture was plated onto Medium 2 plates. Plates were incubated at 60 °C for 48 h. Single colonies on these plates were then inoculated into 5 mL of Medium 2, supplemented with 1% crude oil, and grown on a rotary shaker for 48 h. The culture was then extracted with ethyl acetate and subjected to GC analysis.

### Strain identification

The TIANamp Bacteria Kit (Tiangen, Beijing, China) was used to isolate bacterial chromosomal DNA following the manufacturer’s protocols. The isolated genomic DNA was then used as template to amplify the 16 S rRNA gene via PCR, using the primers listed in Table S1 (see Supplementary Materials). The resulting PCR products were ligated into pGM-Simple-T Fast Vector (Tiangen, Beijing, China), and the resulting plasmids were isolated using the TIANprep Mini Kit (Tiangen). The 16 S rRNA gene fragment from the plasmid was sequenced and analyzed using BLAST with the NCBI nucleotide database.

### Preparation and analysis of surfactin using high-performance liquid chromatography

Surfactin was extracted from the broth of *B. subtilis* GZ6 and *B. subtilis* GZ6 (*pg3srfA*) as previously described (Li et al. 2015). High-performance liquid chromatography (HPLC) analysis was performed using an LC-20 A system (Shimadzu, Kyoto, Japan) equipped with a UV detector. The analytical column was a GL C18 column (25 mm × 4.6 mm, 5 μm; GL Sciences, Kyoto, Japan).

The mobile phase consisted of a mixture of acetonitrile, water, and trifluoroacetic acid (85:15:0.1) at a flow rate of 0.8 mL/min. Surfactin was detected at a wavelength of 205 nm and its concentration was quantified using a standard curve for surfactin.

### Gas chromatography analysis and determination of the alkane degradation rate

Analysis was performed on an Agilent 7890 gas chromatography (GC) system with a flame ionization detector, (Agilent, Santa Clara, CA, USA) equipped with an HP-5 column (30 m × 0.25 μm; Agilent). GC conditions were as follows: injection and detection temperatures were set to 280 °C, the initial oven temperature was set to 50 °C, and increased to 300 °C at a rate of 9 °C/min. The hydrogen and air flow rates were 35 and 400 mL/min, respectively, and the split ratio was set to 10:1. The alkane degradation rate (ADR) was calculated using the following formula:

1 $$ADR = \frac{{{P_2} - {P_1}}}{{{P_2}}} \times 100\% ,$$

where *P2* is the total peak area of the alkane control on GC, and *P1* is the total alkane peak area of the alkane-degrading strain.

### Gas chromatography-mass spectrometry analysis

Gas chromatography-mass spectrometry (GC–MS) analyses were performed on an Agilent GC–MS system (7890 A gas chromatograph and 5975 C mass-selective detector; Agilent Technologies, Santa Clara, CA, USA) equipped with a DB-WAX column (30 m × 0.25 μm, Agilent Technologies). The GC conditions were as follows: split injection (injector temperature 230 °C, split 1/5); oven temperature, programmed from 35 °C (held for 3 min) to 47 °C at 5 °C/min, then to 100 °C at 25 °C/min, then to 145 °C at 2.5 °C/min, and then to 200 °C (held for 5 min) at 25 °C/min; the post-injection dwell time was 0.04 min; carrier gas was He and the and flow rate was 1.0 mL/min; interface temperature was 160 °C; injection volume was 0.2 µL. The mass spectrometry was used in electron impact ionization mode, with an electron energy of 70 eV, an ion source temperature of 230 °C, and a quadrupole temperature of 150 °C. Data were acquired in full-scan (m/z 10–500) mode. Other GC–MS conditions were the same as described in Ai et al. (2014). Data were acquired and analyzed using Enhanced ChemStation (version E.02.00.493, Agilent Technologies). Peaks were identified by comparing volatile sample mass spectra with spectra in the NIST08 Mass Spectral Database of the National Institute of Standards and Technology.

### Routine PCR amplification

PCR amplification was performed using Tsingke PCR Master Mix (Tsingke, China) according to specifications (PCR amplification speed, 1 kb/10 sec). The PCR cycling protocol included an initial denaturation step at 95 °C for 5 min, followed by 30 cycles at 95 °C for 1 min, 53 °C for 1 min, and 72 °C for a specified time (calculated via the DNA fragment length). A final extension step at 72 °C was then performed for 10 min.

### Promoter replacement by single-cross homologous recombination

PCR was used to amplify a 2 kb sequence upstream of the *srfA* promoter region with primers P1 and P2 (provided in Table S1, see Supplementary Materials). An artificial *PG3* promoter was introduced at the 5’ end of the 2 kb DNA fragment using primers P3–P6 (listed in Table S1, see Supplementary Materials) via Gibson assembly (Gibson 2011). The resulting hybrid gene construct (2.1 kb) was then ligated into the RepB site of the pMA5 plasmid (Novogen). The final plasmid construct was designated as pMA5cm*PG3SrfA*. *B. subtilis* GZ6 competent cells were prepared following a previously described method (Xue et al. 1999). The pMA5cm*PG3SrfA* recombinant plasmids were then electroporated into *B. subtilis* GZ6 competent cells using a MicroPulser Plus electroporator (Bio-Rad, Richmond, CA, USA) with settings of 2.5 kV voltage, two pulses, and 2.5 ms time constant. The electroporation was performed in 0.2 cm cuvettes. After electroporation, cells were plated on LB agar containing 30 µg/mL kanamycin. Colony PCR and sequencing were utilized to identify emerging clones. The confirmed strain was named *B. subtilis* GZ6 (*pg3srfA*).

### Plasmid constructions and expression of the LadA gene in *E. Coli* or *G. Stearothermophilus*

The *ladA* gene (1.3 kb, GenBank: ABO68832) was synthesized by Genscript Co (Nanjing, China). The gene was ligated into the expression vector pET30a between the *Nde*I and *Xho*I sites by DNA assembly. This ligation resulted in the recombinant plasmid pET*ladA*, which was then transformed into chemically competent *E. coli* BL21(DE3) cells. A colony of positive transformants was selected and inoculated into 5 mL of Luria-Bertani (LB) medium supplemented with kanamycin. The culture was incubated at 37 °C under shaking until the optical density at 600 nm (OD_600_) reached 0.8. Protein expression was induced by adding isopropyl-β-D-thiogalactoside to a final concentration of 1 mM. After 12 h of induction, cells were harvested by centrifugation at 8,000 RPM for 10 min.

The plasmid for expression in *Geobacillus* strains was constructed by amplifying and inserting a 100 bp *PnppT12* constitutive promoter upstream of the *ladA2mu* gene (Xue et al. 1999). The resulting hybrid gene, *PpladA2mu* (1.4 kb), was then cloned into the *Hind*III site of the pIM1773 plasmid (Addgene, Cambridge, MA, USA) using primers P9–P12 and Gibson assembly. The final plasmid was designated as *pIMPpladA2mu*, which was then transformed into *G. stearothermophilus* competent cells as previously described (Xue et al. 1999). The cells were cultured on Medium 2 plates at 60 °C overnight. Single clones were then inoculated into C18 medium and incubated at 60 °C and 220 RPM for 36 h.

### Molecular docking and in silico mutagenesis

The catalytic site of the LadA protein (PDB ID: 3B9O) was docked with an 18-carbon alkane molecule using Discovery Studio 2019 software (Accelrys, San Diego, CA, USA). The ligand was docked, and interaction energies were calculated using the CDOCKER docking algorithm. Protein and ligand structures were parameterized using the CHARMm force field. The ligand conformations that ranked highest were clustered using a 2.0 Å root-mean-square deviation cut-off and scored based on the CDOCKER interaction energy. The global structure with the lowest binding energy was chosen for further analysis.

Computational site-directed mutagenesis was performed using the Calculate Mutation Energy (Binding) protocol in Discovery Studio 2019 to examine the role of binding pocket residues in complex stabilization. The final docked complexes were energy-minimized using the CHARMm force field and the Smart Minimizer algorithm. *In silico* mutagenesis was performed by calculating the free binding energy of the docked complex. To estimate the effect of individual mutations on complex binding, each residue in the binding pocket was mutated to 19 different amino acids. The mutation binding energy was then calculated as ΔΔG_mut_ = ΔΔG_bind_(mutant) - ΔΔG_bind_(wild-type), where ΔΔG_mut_ represents the mutation energy and ΔΔG_bind_ represents the difference in free energy between bound and unbound states.

### Construction of NNK saturation mutant libraries and high-throughput screening of mutants

NNK saturation mutant libraries were created through NNK saturation mutagenesis (Kretz et al. 2004) using the primers listed in Table S2 (P13–P34, see Supplementary Materials). Mutations in clones were confirmed by DNA sequencing.

The library clones were transferred to 96-well plates, each containing 100 µL of LB medium supplemented with 50 µg/mL kanamycin, and incubated at 37 °C for 12 h under shaking at 200×g. Protein expression was induced by adding isopropyl-β-D-thiogalactoside to a final concentration of 1.0 mM. After a 12-h induction period, cells were harvested by centrifugation at 4,000×g for 10 min. The cell pellets were then suspended in 100 µL of chilled 50 mM Tris-HCl buffer (pH 7.5) containing 10 mg/mL lysozyme. Following 30-min incubation and subsequent centrifugation, the resulting supernatants were transferred to fresh 96-well plates for library screening. The reaction plate was prepared with 120 µL of 50 mM Tris-HCl buffer (pH 7.5), 1.5 mM octodecane, 1.5 mM NADPH, 1.5 mM MgSO_4_, and 0.015% (w/v) Triton X-100 in each well. Next, 30 µL of the LadA mutant library supernatant was added to each well. The plates were incubated at 60 °C for 10 min under shaking. NADPH consumption was determined by measuring the absorbance at 360 nm using a BioTek Synergy 2 Multi-Mode Microplate Reader (BioTek, Beijing, China). A sample without octodecane was used as background control.

### Purification of his-tagged proteins on nickel-chelating columns

Cells grown in a 500 mL expression culture were suspended in 40 mL binding buffer (50 mM NaH_2_PO_4_, 300 mM NaCl, and 10 mM imidazole at pH 8.0) and lysed by sonication in an ice bath for 20 min at 200 W. The supernatant was then applied to a 1 mL Novagen His-Bind gravity flow column equilibrated with 20 mL Ni-NTA binding buffer. The column was washed with 20 mL wash buffer (50 mM NaH_2_PO_4_, 300 mM NaCl, and 20 mM imidazole at pH 8.0). The His-tagged protein was eluted with 10 mL elution buffer (50 mM NaH_2_PO_4_, 300 mM NaCl, and 200 mM imidazole at pH 8.0). To remove imidazole from the elution buffer, the eluate was applied to a 5 mL HiTrap desalting column (GE Healthcare, USA) and eluted with 50 mM Tris-HCl buffer.

### Activity assays of purified and crude LadA and determination of kinetic constants

The reaction mixture was composed of 1 mM octodecane, 0.015% (w/v) Triton X-100, and 10 mM NADPH in 50 mM Tris-HCl buffer (pH 8.0) with a volume of 200 µL. To ensure homogenization, the mixture was sonicated for 1 min. Subsequently, 100 µl of the same buffer containing 0.1 µg of purified enzyme was added. Reactions were initiated by incubation at 60 °C for 10–20 min and terminated by extraction with an equal volume of chloroform. NADPH consumption was determined following the method as described (Dubbels et al. 2007). *K*_m_ and *V*_max_ values were calculated using Lineweaver-Burk plotting plots. Reduced flavin mononucleotide (FMNH_2_) consumption was calculated by measuring the increase at A_455_ during alkane oxidation (Uetz et al. 1992) using a Shimadzu PharmaSpec UV-1700 UV-VIS spectrophotometer (Tokyo, Japan). A total of 10–30 µmol FMN was added to the reaction mixture and the initial reaction rate was measured spectrophotometrically. Michaelis-Menten analyses for FMN were then performed with initial velocities using GraphPad Prism v5.0 (GraphPad Software, Boston, MA, USA) to calculate the Michaelis-Menten constant (*K*_m_).

Alkane consumption was analyzed using GC, as described above. Enzyme activity was defined as the amount that oxidizes 1 µmol of NADPH or 1 µmol of octodecane per min. To measure expressed alkane monooxygenase activity in *G. stearothermophilus* GZ178, cells from a 50 mL expression culture were suspended in 4 mL of Tris-HCl buffer (50 mM, pH 8.0) and disrupted by sonication for 20 min at 200 W in an ice bath. The resulting supernatant (100 µL) was added to the same reaction mixture under identical conditions as described for purified LadA.

### Determining the dewaxing rate by crude oil weight loss test

In a flask containing 40 g of crude oil (M1), 40 mL of microbial dewaxing agent culture was added, while in the control flask (M2), the culture was replaced by 40 mL of sterile water. The empty weights of the flasks were measured and recorded as M1_0h_ and M2_0h_, respectively. The two flasks were incubated at 60 °C and 120 RPM for 72 h. After incubation, they were placed in an incubator at 35 °C for 90 min to allow the oil to solidify. The flasks were then inverted and incubated at 40 °C for 60 min. Finally, the total weights of flasks were measured again and recorded as M1_72h_ and M2_72h_, respectively. Each experiment was replicated three times. The dewaxing rate (DWR) was calculated using the following formula.

2 $$DWR = \frac{{\Delta {M_2} - \Delta {M_1}}}{{\Delta {M_2}}} \times 100\% ,$$

where ∆ M1 = M1_72h_ - M1_0h_; ∆ M2 = M2_72h_ - M2_0h_.

### Optimizing the composition of the microbial dewaxing agent

Individual colonies from strain-preserving plates were inoculated into 5 mL of medium and grown on a rotary shaker at 40–60 °C and 220 RPM for 24 h. The cultures were then transferred into fresh 50 mL medium and further grown under the same conditions for 48 h. *B. subtilis* GZ6 (*pg3srfA*) was cultured in Medium 1 at 40 °C, while *G. thermodenitrificans* GZ156 and *G. stearothermophilus* GZ178 (*pIMPpladA2mu*) were cultured in Medium 2 at 60 °C. The resulting cultures were mixed at a specific ratio and subjected to crude oil weight loss test (Yi 2015).

## Results and discussion

### Bacterial screening and strain identification

**Fig. 1:**
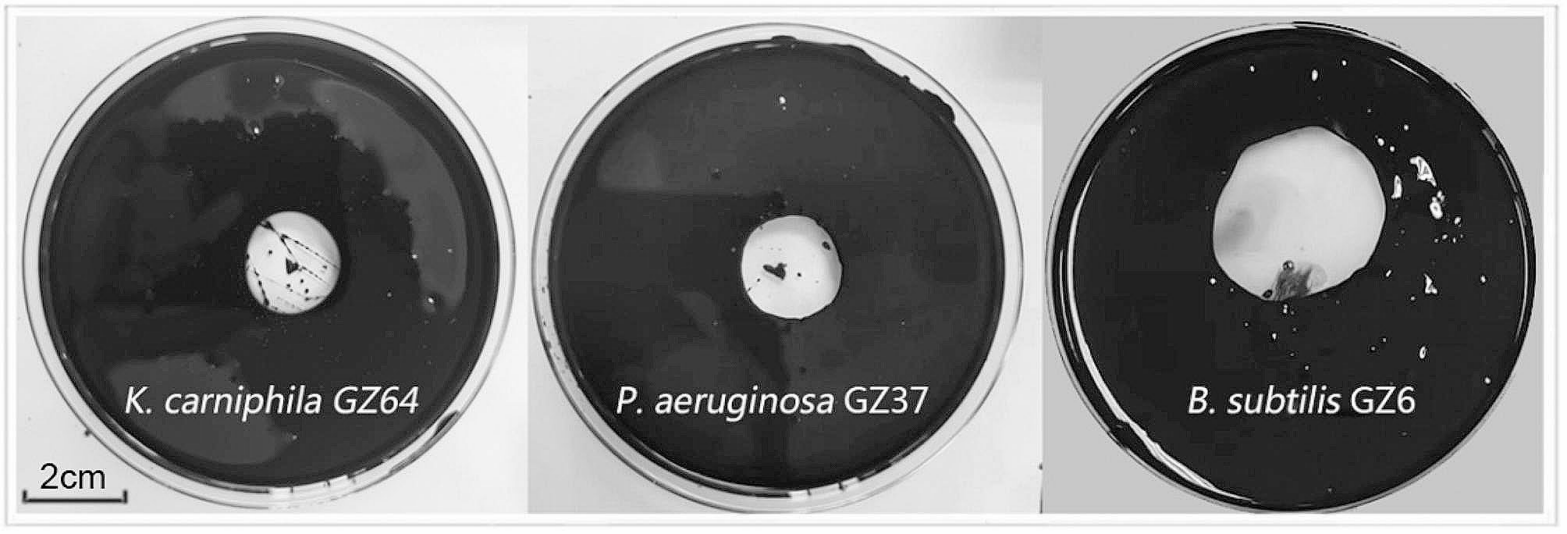
Positive strains in the oil spreading test. Average diameters of oil discharge rings: *Kocuria carniphila* GZ64, 1.7 ± 0.1 cm, *Pseudomonas aeruginosa* GZ37, 1.9 ± 0.1 cm, and *Bacillus subtilis* GZ6, 3.5 ± 0.2 cm; Each experiment was performed in triplicate

Oil wells are typically located at depths of around 2000 m, resulting in temperatures of approximately 60–70 °C. Therefore, initial screenings for bacteria capable of producing biosurfactants and degrading crude oil were conducted at 60 °C. However, no biosurfactant-producing strains were identified through oil spreading tests at this temperature. Subsequently, screening was conducted at gradually decreasing temperatures, and biosurfactant production was observed in three strains when cultured at 40 °C. These three bacteria that produced biosurfactants were identified as *B. subtilis*, *Pseudomonas aeruginosa*, and *Kocuria carniphila* through 16 S rRNA gene sequencing (Table 1). Among these three strains, *B. subtilis* GZ6 exhibited the most effective performance in oil spreading assays (Fig. 1). Currently, the most commonly used microbial species for biosurfactant production in microbial dewaxing agents is *Pseudomonas aeruginosa* (Soares Dos Santos et al. 2016). This bacterium is known for its high-yield production of rhamnolipids, and several strategies have been proposed to further enhance rhamnolipid production in this strain (She et al. 2011). However, despite being obtained in the initial round of screening, this strain was ultimately excluded from further experimentation because of its potential risk to public health and safety. The screening process in this study did not yield any thermophilic microorganisms capable of biosurfactant synthesis.

**Table 1:** Bacteria with crude oil degrading ability or biosurfactant productivity

Strain name	Medium	Culture temperature (°C)	Strain function
*Bacillus subtilis GZ6*	1	40	Biosurfactant producing
*Pseudomonas aeruginosa GZ37*	1	40	Biosurfactant producing
*Kocuria carniphila GZ64*	1	40	Biosurfactant producing
*Brevibacillus agri GZ143*	2	60	Crude oil degrading
*Geobacillus thermodenitrificans GZ156*	2	60	Crude oil degrading
*Geobacillus stearothermophilus GZ178*	2	60	Crude oil degrading
*Geobacillus kaustophilus*	2	60	Crude oil degrading
*Geobacillus subterraneus*	2	60	Crude oil degrading
*Corynebacterium thermophilum*	2	60	Crude oil degrading

**Fig. 2:**
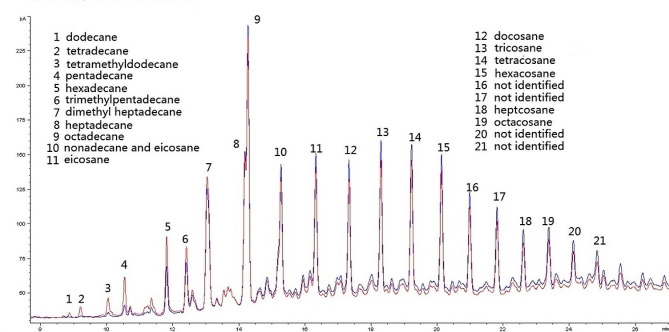
Gas chromatography-mass spectrometry (GC–MS) analysis of alkane degradation. The degradation experiment was performed in triplicate and no significant differences were observed between the obtained GC–MS chromatograms. Blue line, *G. thermodenitrificans* GZ156 degradation, Red line, *G. stearothermophilus* GZ178 degradation

During the screening process for crude oil degrading bacteria, six strains capable of degrading alkanes were obtained and identified through sequencing of their 16 S rRNA gene (Table 1). GC–MS analysis was used to determine the substrate specificities and degradation rates of these strains, with *G. thermodenitrificans* GZ156 and *G. stearothermophilus* GZ178 exhibiting the highest rates at approximately 47.6% and 30.1%, respectively (Fig. 2 and Table S3). These two strains have different alkane substrate specificities: *G. thermodenitrificans* GZ156 degrades alkanes with low boiling points, such as dodecane, tetradecane, and pentadecane, while *G. stearothermophilus* GZ178 prefers alkanes with high boiling points, such as heptacosane and octacosane (Fig. 2 and Table S3).

### Enhancing *B. subtilis* biosurfactant productivity by promoter replacement

**Fig. 3:**
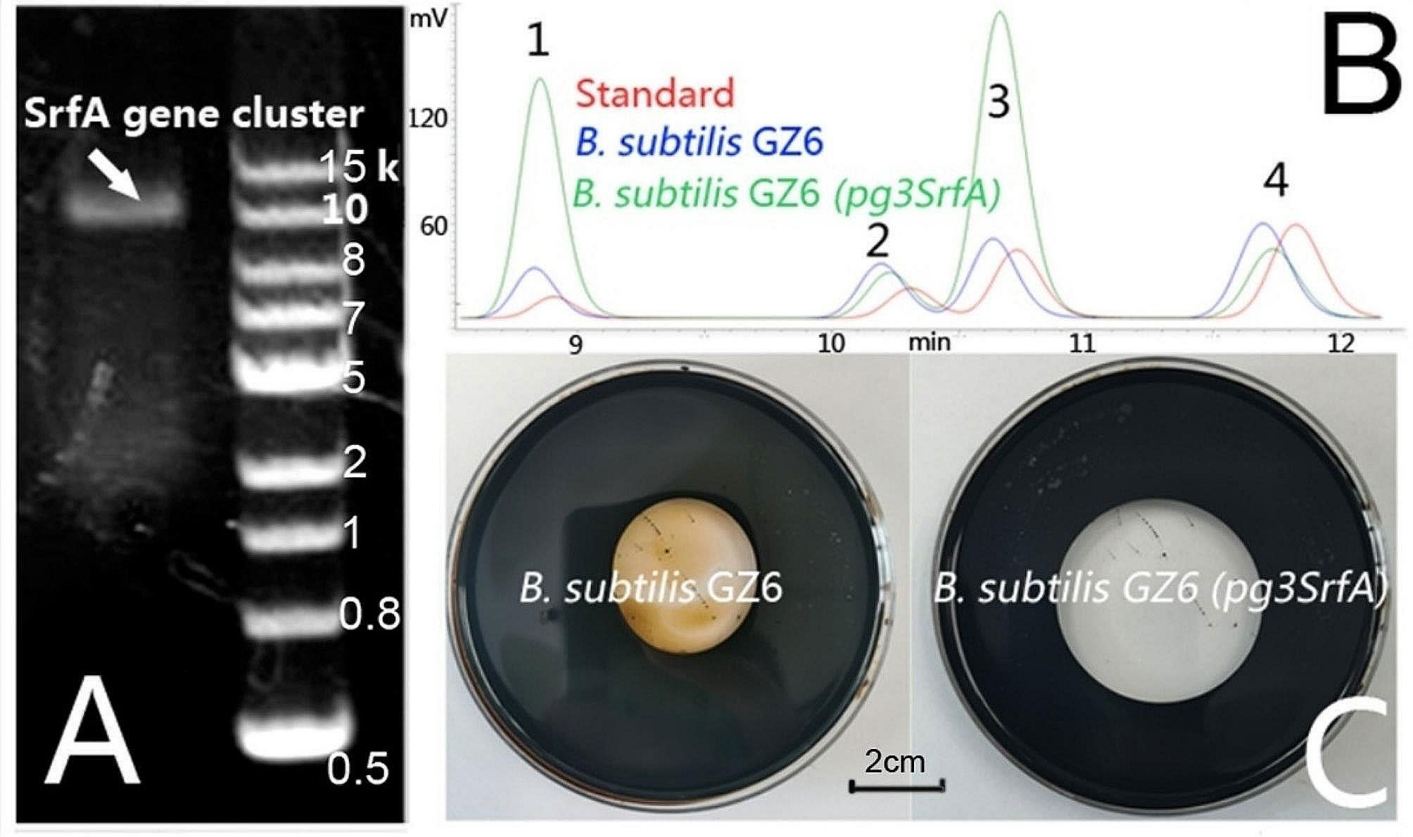
Enhancing the *B. subtilis* GZ6 surfactin productivity by promoter replacement. **A**, PCR amplification of the *srfA* gene cluster from the *B. subtilis* GZ6 genome. **B**, Surfactin produced by *B. subtilis* GZ6 (blue); and *B. subtilis* GZ6 (*pg3srfA*) (green). Four peaks (1, 2, 3, and 4) were observed for the surfactin standard (red) when analyzed by HPLC. **C**, Oil spreading tests for *B. subtilis* GZ6 and *B. subtilis* GZ6 (*pg3srfA*). Average diameters of oil spreading rings (each experiment was performed with three replicates), *B. subtilis* GZ6, 3.5 ± 0.1 cm; *B. subtilis* GZ6 (*pg3srfA*), 4.1 ± 0.2 cm

HPLC analysis using purchased standards (Fig. 3B) identified the biosurfactant produced by *B. subtilis* GZ6 as surfactin (Hsieh et al. 2008). Surfactin is typically composed of four cyclic lipopeptides, with chain lengths ranging from C13 to C16, that differ slightly in their amino acid sequences. The proportion of these four cyclic lipopeptides in the surfactin mixture varies among different *B. subtilis* strains (Jiao et al. 2017).

Native *B. subtilis* strains typically have low surfactin titers. To increase surfactin yield in these strains, several strategies were employed, including promoter replacement, which was identified as the most effective and simple approach (Li et al. 2015; Jiao et al. 2017). Surfactin is produced by surfactin synthase (SrfA), which is encoded by the seven-module non-ribosomal peptide synthetase gene cluster (*srfA*). When using the native *srfA* promoter, the transcription levels of *srfA* were low. However, the transcription of *srfA* in *B. subtilis* THY-7 could be initiated effectively by using a modified hybrid *Pg3* promoter (Jiao et al. 2017). The *B. subtilis* GZ6 *srfA* gene cluster identified by PCR amplification and sequencing showed 99.8% homology to the *B. subtilis* THY-7 *srfA* gene cluster (Fig. 3A). Subsequently, the *srfA* promoter was replaced with the *Pg3* promoter through the recombination of single-cross homologous (Jiao et al. 2017). The genetically modified *B. subtilis* GZ6 strain was confirmed through PCR and sequencing.

The production of surfactin by *B. subtilis GZ6* (*pg3srfA*) increased to 2.32 g/L, compared to the production of 0.33 g/L of the wild-type strain. The modified strain of *B. subtilis* GZ6 (*pg3srfA*) produced significantly larger spreading rings in oil spreading assays (Fig. 3C). Additionally, *B. subtilis* GZ6 (*pg3srfA*) exhibited a growth curve comparable to that of *B. subtilis* GZ6, indicating that the replacement of the promoter did not affect cell growth (Fig. S2, A, Supplementary Materials).

### LadA expression and engineering in *E. Coli*

**Fig. 4:**
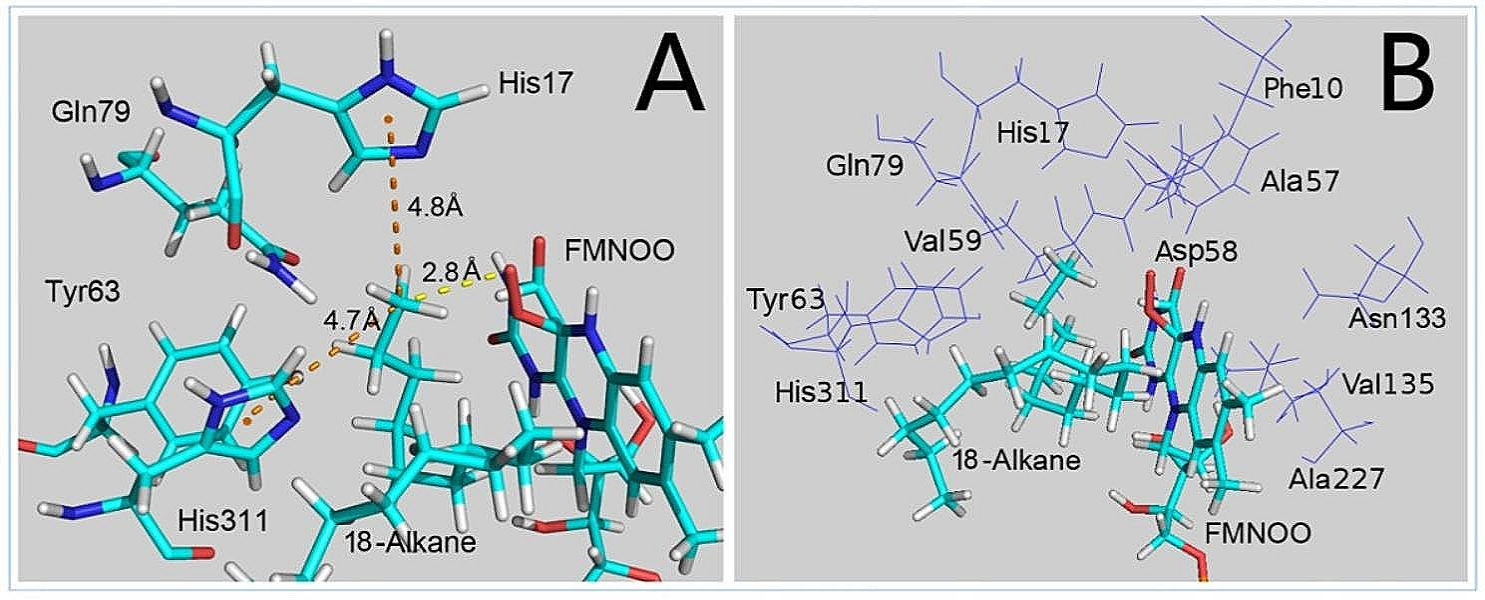
Molecular docking (**A**) and residues of the LadA binding pocket (**B**). **A**, The distance between the oxygen atom of flavin mononucleotide (FMNOO) and the alkane terminal carbon is indicated by yellow dashed lines, while Pi-Alkyl interactions are indicated by orange dashed lines. **B**, Residues constituting the LadA binding pocket are represented by lines; C18 alkane and FMNOO are displayed as sticks

The high viscosity of RO is mainly the result of long-chain alkanes, also known as wax, with a carbon chain length of 18 or longer. The microbial dewaxing process targets these long-chain alkanes as they pose the primary obstacle to heavy crude oil recovery (Adlan et al. 2020). Based on preliminary screening results (Table S3), *G. thermodenitrificans* GZ156 did not degrade octadecane, while *G. stearothermophilus* GZ178 showed poor ADR on the substrate. Therefore, octadecane was selected as the target substrate for this research.

A previous study by Feng et al. (2007) illustrated the metabolic pathway for long-chain alkanes in *G. thermodenitrificans*. The pathway involves the utilization of an alkane monooxygenase (LadA), which activates alkanes to corresponding primary alcohols. LadA can degrade alkanes ranging from C15 to C36, with hexadecane being the optimal substrate and octadecane being the sub-optimal substrate. The first enzyme in a metabolic cascade usually catalyzes the rate-limiting step (Chen et al. 2008). It is worth noting that alkane monooxygenases generally have lower catalytic efficiencies than enzymes such as hydrolases. Increasing the copy number of the *ladA* gene in *G. thermodenitrificans* GZ156, particularly a version of LadA that was enhanced through protein engineering, may have the potential to improve the alkane metabolic cascade of the strain, especially in octadecane degradation, and subsequently enhance the wax removal rate.

Initial PCR amplification of the *ladA* gene from the total DNA of *G. thermodenitrificans* GZ156 or *G. stearothermophilus* GZ178 was unsuccessful. As previously reported, the *ladA* gene is located on a native plasmid (Feng et al. 2007), and it is likely that this plasmid is absent in these two strains. Despite this absence, their ability to degrade alkanes suggests the presence of an additional, as yet unidentified alkane hydroxylase system that also produces alcohol intermediates (Smits et al. 2002). To improve the alkane degrading capacity of these strains, the introduction of an improved LadA enzyme is a more promising option. However, protein engineering of LadA requires efficient gene manipulation and effective expression systems, that are lacking in *Geobacillus* species. The *ladA* gene was well expressed in *E. coli* BL21(DE3) under the T7 promoter (Fig. S1). Therefore, the initial engineering for LadA was conducted in the *E. coli* host.

Although the structure of LadA has been resolved, its catalytic mechanism has not yet been identified. However, LadA belongs to the SsuD subfamily within the bacterial luciferase family (Chen et al. 2008), and the catalytic mechanism of this subfamily has been well elucidated (Armacost et al. 2014). Therefore, the SsuD catalytic hypothesis was applied to propose a catalytic initiation mechanism for LadA. According to this hypothesis, the alkane terminal carbon is attacked by the distal oxygen atom of the C4a-hydroperoxyflavin of flavin mononucleotide. Protein-ligand docking was performed based on this hypothesis. The analysis focused on the lowest energy configuration among docking poses. In this configuration, the flavin mononucleotide distal oxygen atom was 2.8 Å away from the alkane terminal carbon. The terminal carbon was stabilized by two pi-alkyl interactions (Ozawa et al. 2008) with the imidazole rings of His17 and His311 (Fig. 4A).

According to the protein-ligand docking mechanism, the binding energy of the substrate ligand is typically positively correlated with catalytic efficiency (Londhe et al. 2019). To enhance substrate binding, virtual saturation mutagenesis was conducted and 25 residues within 5 Å of the docked substrate were analyzed. The changes in free energy (ΔG) were compared, showing that the Ala57His mutant presented the most pronounced reduction in binding energy (-5.0 kJ/mol). Mutations at the Phe10, His17, Ala57, Asp58, Val59, Tyr63, Gln79, Asn133, Val135, Ala227, and His311 sites resulted in ΔG values ranging from − 5.0 to -2.3 kJ/mol (Fig. 4B, Table S2, Supplementary Materials), suggesting that these mutations were beneficial. Mutants of other sites showed ΔG values ranging from − 2.3 to 2.9 kJ/mol, indicating either neutral or damaging effects (Spassov and Yan 2013).

The residues listed in Table S2 (Supplementary Materials) were applied for NNK saturation mutagenesis. The resulting mutant libraries were screened using high-throughput methods. A mutant with increased activity, namely Phe10Leu, was verified through GC analysis. NNK saturation mutagenesis was then performed on Phe10Leu at the other sites listed in Table S2. After screening and verification, the double mutant Phe10Leu/Asn133Arg (harboring pET*ladA2mu*) was identified. The catalytic efficiency of this mutant was 11.7 times higher than that of the wild-type enzyme (Table 2). Table 2 shows that both single and double mutants had significantly lower *K*_m_ values for the substrate compared to the wild-type strain, indicating an increased affinity between enzyme and substrate. The affinity between the single mutant and FMN remained unchanged, while the affinity between the double mutant and FMN decreased slightly. Overall, the mutation of the enzyme at these two sites has a minimal effect on the binding of coenzyme FMN to the enzyme. However, the mutation significantly increased the affinity between substrate and enzyme, thereby greatly improving the catalytic efficiency of the enzyme.

**Table 2:** Catalytic constants for LadA and mutants

Enzyme	K_m_	k_cat_	k_cat_/K_m_	K_mFMN_ [µM]
	[mM]	[min^− 1^]	[mM^− 1^·min^− 1^]	
LadA	10.36 ± 1.6	1.78 ± 0.2	0.17	3.3 ± 0.2
LadAF10L	6.98 ± 0.5	2.16 ± 0.3	0.31	3.1 ± 0.3
LadAF10L/N133R	2.04 ± 0.2	4.42 ± 0.6	2.17	3.9 ± 0.3

As previously reported, LadA was engineered through random and site-directed mutagenesis with the goal to enhance hexadecane oxidation (Dong et al. 2012). However, the best mutant obtained (F146N/N376I) differed significantly from the Phe10Leu/Asn133Arg mutant. This difference may be the result of the use of different substrates (hexadecane versus octadecane) for screening mutant libraries, resulting in variations in the binding of active sites and substrates. The use of random mutagenesis or virtual mutagenesis may have led to two different directed evolutionary pathways for LadA.

### Expression of *ladA2mu* in *G. Stearothermophilus* GZ178 and octodecane transformation

After obtaining the *ladA2mu* gene from the *E. coli* host, attempts were made to express *ladA2mu* in *G. thermodenitrificans* GZ156 and *G. stearothermophilus* GZ178. However, no plasmid could be employed to express this gene in *G. thermodenitrificans* GZ156. The *ladA2mu* gene was successfully expressed in *G. stearothermophilus* GZ178, which was harboring plasmid *pIMPpladA2mu*. The relevant protein band was not detected in the sodium dodecyl sulfate-polyacrylamide gel electrophoresis analysis, suggesting low expression levels (Fig. S1, Supplementary Materials). However, the cells expressing the plasmid showed a significant increase in octodecane conversion compared to cells carrying the empty plasmid (from 25.0 to 75.3%, Fig. S3, Supplementary Materials). In addition, *G. stearothermophilus* GZ178 (*pIMPpladA2mu*) cells exhibited an expanded substrate spectrum toward short-chain alkanes, such as pentadecane and hexadecane (Table S3, Supplementary Materials). The growth curve of *G. stearothermophilus* GZ178 (*pIMPpladA2mu*) was comparable to that of *G. stearothermophilus* GZ178 (Fig. S2, B, Supplementary Materials). These data suggest that plasmid expression did not affect cell growth.

### Optimization of the constitution of the microbial dewaxing agent

**Fig. 5:**
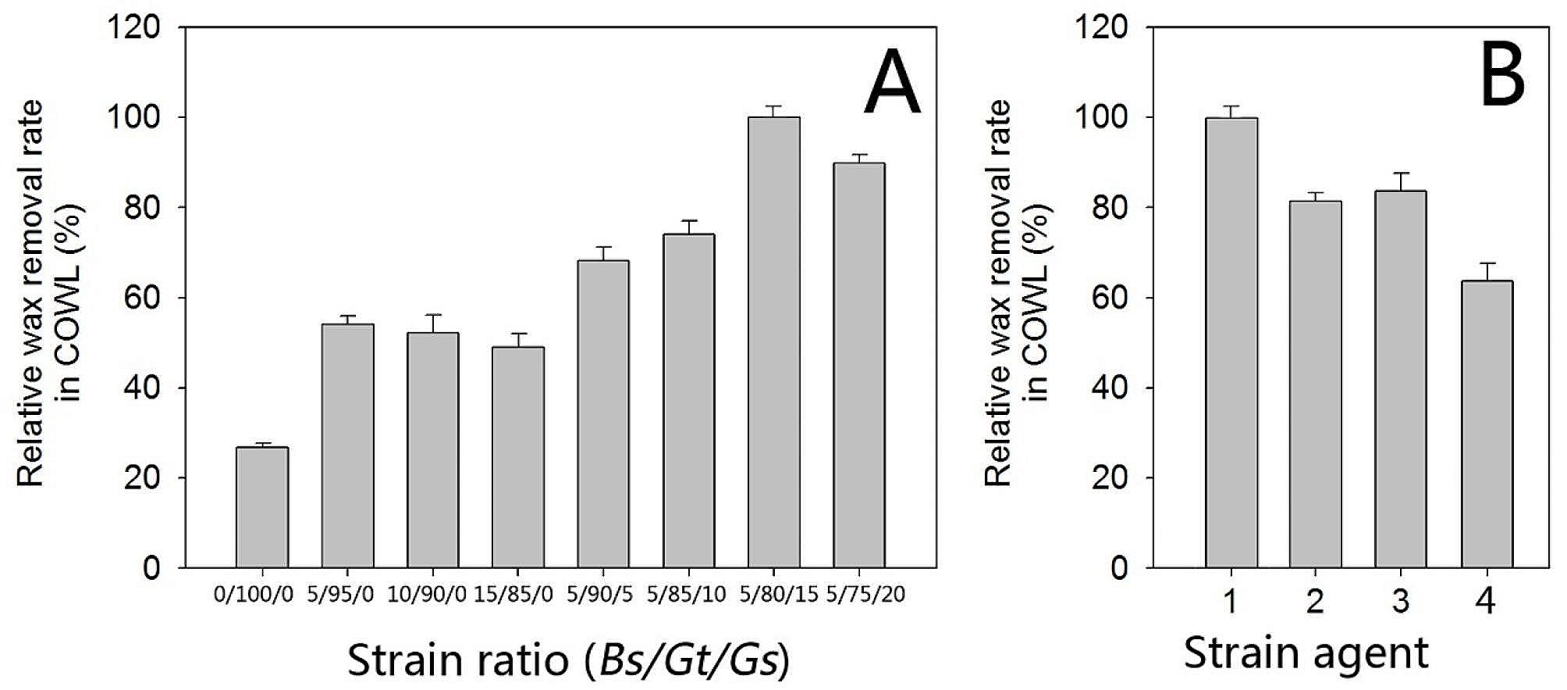
Optimization of the constitution for microbial dewaxing agents. Each experiment was performed in triplicate. **A**, Bs, *B. subtilis* GZ6 (*pg3srfA*); Gt, *G. thermodenitrificans* GZ156; Gs, *G. stearothermophilus* GZ178 (*pIMPpladA2mu*). The 100% relative wax removal rate is 60.3 ± 3.2%. The OD_580_ of the cultures are 8.0 ± 0.1 for Bs, 3.0 ± 0.2 for Gt, and 6.0 ± 0.1 for Gs. **B**, All strain ratios are the same as 5/80/15 (Bs/Gt/Gs). 1, Same strain constitution ratio as the 5/80/15 agent in A; 2, Gs wild type strain (OD_580_ = 6.0) in the agent; 3, Bs wild type strain (OD_580_ = 8.0) in the agent; 4, All strains are wild type strains (Bs, OD_580_ = 8.0, Gt OD_580_ = 3.0, Gs OD_580_ = 6.0)

This study initially used the two strains of *B. subtilis* GZ6 (*pg3srfA*) and *G. thermodenitrificans* GZ156 in the agents. As shown in Fig. 5A, the DWR of the single strain agent (*G. thermodenitrificans* GZ156 alone) was only 50% compared to that of the double strain agent; therefore, the biosurfactant played a critical role in wax removal. Previous reports have shown that *G. thermodenitrificans* and *G. stearothermophilus* have different substrate preferences for carbon chain length (Lin et al. 2019). While *G. thermodenitrificans* prefers short-chain alkanes, such as dodecane, *G. stearothermophilus* prefers long-chain alkanes, such as octacosane (Fig. 2 and Table S3). It is proposed that the dewaxing rate could be improved by broadening the substrate spectrum of the microbial dewaxing agent. Therefore, *G. stearothermophilus* GZ178 (*pIMPpladA2mu*) was also introduced into the dewaxing agent. Compared to the wild-type strain, *G. stearothermophilus* GZ178 (*pIMPpladA2mu*) cells have an extended substrate spectrum (Table S3, Supplementary Material). The optimal microbial agent had the highest DWR when all three strains were mixed at a ratio of 5:80:15 (Fig. 5A). Figure 5B shows that the increase in surfactin production for *B. subtilis* GZ6 and the expression of *ladA2mu* in *G. stearothermophilus* GZ178 both contributed to the increase in DWR. DWR decreased when either modified strain was replaced by its wild-type strain. The complex composed of the native strains showed a DWR that was only 65% of that of the optimized composition (Fig. 5B).

The microbial dewaxing agent operates through two main mechanisms: inhibiting the growth of wax crystals and degrading the existing wax. These dual functionalities rely on the biosurfactant production and wax-degrading activities of microbial strains. In this microbial dewaxing agent, strain *B. subtilis* GZ6 (*pg3srfA*) functions as biosurfactant producer while the other two strains degrade wax. The biosurfactant produced by *B. subtilis* GZ6 was surfactin, which remained stable even at a temperature of ~ 140 °C (Jiao et al. 2017). It is proposed that, during the sedimentation process following inoculation into oil wells, *B. subtilis* in the microbial agent proliferates within the appropriate temperature range at certain depths and continues to produce biosurfactants.

The introduction of *G. stearothermophilus* GZ178 (*pIMPpladA2mu*) into the agent significantly improved DWR for two reasons: First, the substrate spectrum of *G. stearothermophilus* GZ178 (*pIMPpladA2mu*) in alkane degradation was significantly extended by the introduction of a heterologous *ladA2mu* gene. When the strain was incorporated into the microbial agent, the DWR was increased by approximately 35%. Furthermore, although both *Geobacillus* species belong to the same genus, they have different alkane metabolic pathways. *G. thermodenitrificans* metabolizes long chain alkanes by the primary oxidation pathway, in which the C atom at the end of the alkane is first oxidized to alkanol by alkane oxidase, then sequentially converted to aldehyde and fatty acid, and finally enters β-oxidation (Feng et al. 2007). However, *G. stearothermophilus* metabolizes alkanes through secondary oxidation, in which long chain alkanes are oxidized to secondary alcohols and acetic acid. These secondary alcohols are further oxidized to ketones and esters, which then enter the same pathway as in *G. thermodenitrificans* (Liu et al. 2008). This diversity of pathways can effectively mitigate inhibition by intermediate metabolites during the alkane metabolism, resulting in significantly improved wax removal efficiency.

### Biosafety and ethical considerations of microbial dewaxing agents in environmental applications

The use of genetically engineered microbes in environmental applications presents a complex set of challenges and imposes ethical considerations. The microbial dewaxing agent in this study has high biosafety. First, the agent was applied in oil fields with relatively harsh environments and relatively small microbial populations, thus minimizing horizontal transfer of resistance genes. Second, the genetically modified hyperthermophilic bacteria in this agent cannot multiply at room temperature, thus limiting their gene spread to mesophilic microbes. Third, *Bacillus subtilis* is considered a GRAS strain (i.e., Generally Recognized As Safe) and both native and gene modified strains are widely used in food, pharmaceutical, and other fermentation industries (Ejaz et al. 2022).

### Research status and future research directions for microbial dewaxing agents

Currently, most microbial dewaxing agents are composed of multiple bacteria, while only few are composed of single bacteria (Patel et al. 2015). Certain agents may contain non-biological security microorganisms, such as *Bacillus anthracis* (Liu et al. 2014) and *Pseudomonas aeruginosa* (Câmara et al. 2019). The DWRs of these agents vary widely, ranging from 12.3 to 68.2%. Comparing the DWR values of these agents alone does not provide meaningful insight. Crude oil components have different geographic characteristics, especially regarding the amounts of heavy oil and wax contents. Therefore, it is necessary to verify the effectiveness of dewaxing agents through field experiments in oil wells. The microbial dewaxing agent developed in this study has a high dewaxing rate of 60.2% and is environmentally safe. Field tests conducted in several oil wells have presented positive results in terms of dewaxing and increased production (data not shown).

Spore-forming bacteria, such as *Bacillus*, *Geobacillus*, and *Clostridium*, have been utilized as microbial dewaxing agents (Preeti et al. 2019; Zhou et al. 2016). These bacteria are frequently found in deep oil reservoirs and can withstand the prevalent harsh conditions, including nutrient deprivation, extreme temperatures, and high salt concentrations. The microbial dewaxing agent in this study was also developed using spore-producing microorganisms, which allows for a long-lasting effect because of the spores’ resistance to adverse environments. Therefore, bacterial strains capable of forming endospores and degrading petroleum or synthesizing biosurfactants are potential targets for further screening and isolation of microorganisms that remove wax. Similarly, extremophilic microbes have also been examined for microbial dewaxing applications because of their ability to withstand extreme environments. These microbes are either thermophilic, halophilic, or barophilic (Varjani and Upasani 2016; Lin et al. 2019). In addition, genetically engineered microbes have been used for microbial engineered wax removal through techniques such as protoplast fusion, gene knockout, and genome editing. These technologies have the potential to improve the temperature adaptability of microbes (Sun et al. 2017) and increase their biosurfactant productivity (Jiao et al. 2017).

Microbial dewaxing technology reduces reliance on conventional chemicals and lowers the risk of environmental contamination. It uses naturally occurring microorganisms to enhance the oil recovery process, thus reducing the need for harmful chemicals and helping to protect soil and water quality. The use of microbial dewaxing agents in oil recovery can not only reduce resource waste, but also extend the productive life stage of oil fields. Research and practical applications of microbial dewaxing agents have increased because of their potential economic benefits for oil extraction companies as well as for the green and sustainable development of energy extraction technology.

In summary, the ideal microbial dewaxing agent should possess thermophilic, halophilic, and barophilic properties. Such microbes can be isolated from the environment of deep-sea hydrothermal vents (Jin et al. 2019). However, native microbes with superior dewaxing performance are rarely obtained in such harsh reservoir conditions. To overcome this challenge, several methods can be employed. One such method is to customize microbial strains by designing or assembling novel synthetic metabolic pathways for the production of biosurfactants or wax-degrading pathways. Another method is to engineer and express enzymes with strong wax-degrading capabilities in a suitable host. The first step in achieving these goals is to create the appropriate genetic operating system and gene editing tools. The application of synthetic biology technology for microbial dewaxing, which is also the scope of microbial engineered wax removal, has the potential to significantly improve its efficiency.

## Conclusion

In this study, three spore-forming bacterial strains were obtained via screening crude oil-contaminated soil samples. The surfactin titer of strain *B. subtilis* GZ6 was increased by promoter replacement of the surfactin synthase gene cluster (*srfA*). The enhanced alkane monooxygenase double mutant LadAF10L/N133R was generated by site-directed mutagenesis and then expressed in *G. stearothermophilus* GZ178. The final microbial dewaxing agent, consisting of two modified strains (*B. subtilis* GZ6 (*pg3SrfA*) and *G. stearothermophilus* GZ178 (*pIMPpladA2mu*)) and the native strain *G. thermodenitrificans* GZ156, achieved a 35% higher DWR compared to a composition consisting of three native strains.

### Electronic supplementary material

Below is the link to the electronic supplementary material.

Supplementary Material 1

Supplementary Material 2

Supplementary Material 3

Supplementary Material 4

## Data Availability

The datasets generated during and/or analyzed during the current study are available from the corresponding author on reasonable request.
