# Supplementary material for: Development of a microbial dewaxing agent using three spore forming bacteria
Source: Bioresour Bioprocess. 2024 Aug 8;11(1):80. doi: 10.1186/s40643-024-00795-z (PMC11310373; doi:10.1186/s40643-024-00795-z)
Supplement: Supplementary file 1 — Supplementary Material 1 [file 40643_2024_795_MOESM1_ESM.docx]

**Supplementary Materials**

**Development of a microbial wax removal agent using three spore forming bacteria**

Xiaoyan Guo^1,2^, Xutao Zhao^1,2^, Lizhu Li^2^, Jinchang Wang^3^，Haibo Jin^1,2*^, Jianjun Wang^4*^,

^1^ College of New Materials and Chemical Engineering, Beijing Institute of Petrochemical Technology, Beijing, People’s Republic of China

^2^ Beijing Key Laboratory of Fuels Cleaning and Advanced Catalytic Emission Reduction Technology, Beijing, People’s Republic of China

^3^ Institute of Microbiology Jiangxi Academy of Sciences, Nanchang, Jiangxi 330096, P.R. China

^4^ CAS Key Laboratory of Microbial Physiological and Metabolic Engineering, Institute of Microbiology, Chinese Academy of Sciences, Beijing 100101, P.R. China

**Running title**: Development of a microbial wax removal agent


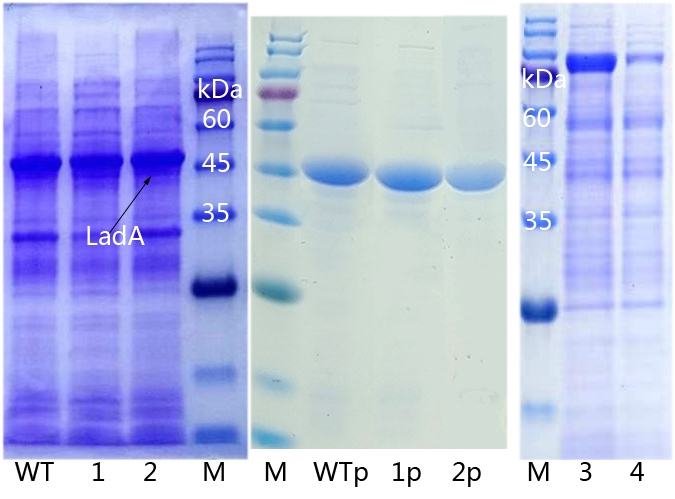


**Fig.S**1 Expressions of LadA and LadA2mu genes in *E. coli* BL21 (DE3) (WT, 1 and 2), purified proteins (WTp, 1p and 2p) and expression of LadA2mu gene in *Geobacillus stearothermophilus* (3 and 4)

WT, wild type LadA; 1, LadAPhe10Leu; 2, LadAPhe10Leu/Asn133Arg (LadA2mu); WTp, purified wild type LadA; 1, purified LadAPhe10Leu; 2, purified LadAPhe10Leu/Asn133Arg (LadA2mu); M, protein marker;

3, *Geobacillus stearothermophilus* with empty *pIM1773* plasmid;

4, *Geobacillus stearothermophilus* harboring *pIMPpladA2mu* plasmid;


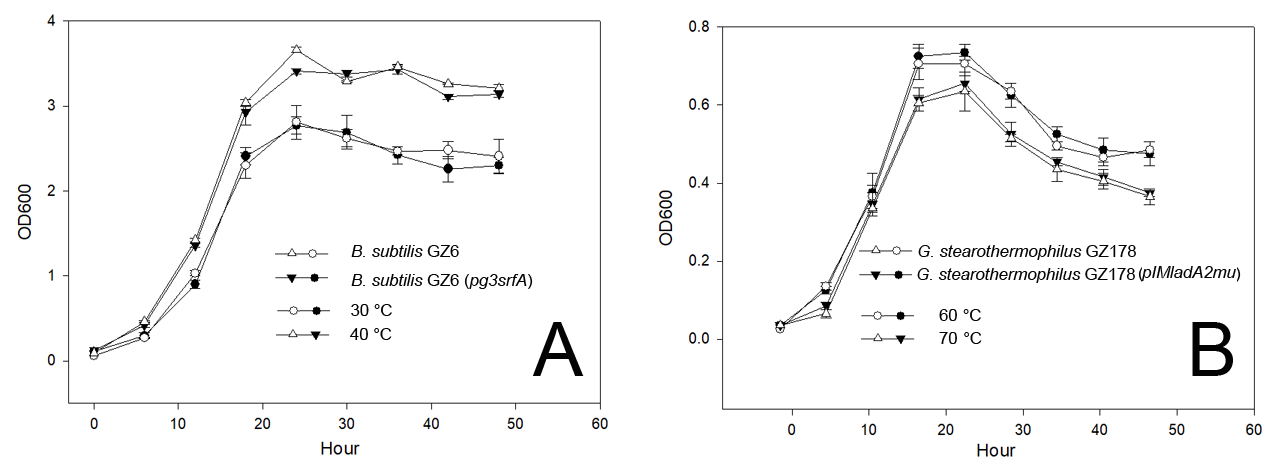


**Fig.S2** Determining growth curves of wild type and genetically modified strains


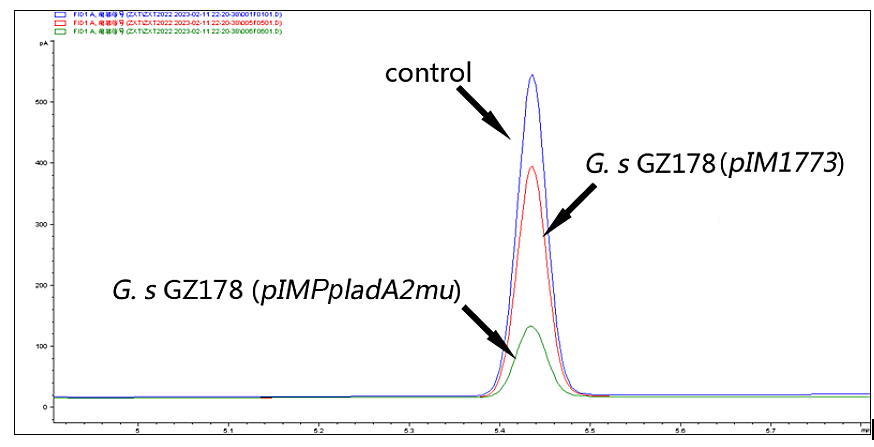


**Fig.S3** The conversion of octodecane for the *G. stearothermophilus* GZ178 cells

Control, octodecane without cells;

*G. s* GZ178 (pIM1773), octodecane conversion of *G. stearothermophilus* GZ178 harboring empty plasmid (conversion 25.5%);

*G. s* GZ178 (pIMladA2mu), octodecane conversion of *G. stearothermophilus* GZ178 harboring pIMladA2mu plasmid (conversion 74.9%);

**Table S1** Primers and promoters used in this work

| **Name** | **DNA-sequence（5’-3’）** | **Description** |
| --- | --- | --- |
| 27F | 5'-AGAGTTGATCCTGGCTCAG-3' | Strain identifying |
| 1492R | 5'-GGYTACCTTGTTACGACTT-3' | Strain identifying |
| P1 | 5’-atggaaataactttttaccc-3’ | srfA upstream, PCR of SrfA cluster |
| P2 | 5’-ttagaaaatttccattaatt -3’ | srfA downstream, , PCR of SrfA cluster |
| P3 | 5’-aattaatggaaattttctaattcaacaaacgggccagttt-3’ | PMA5 plasmid and upstream of PG3 upstream |
| P4 | 5’- GAATCTCCCTTCCAATGTCAAGTGATAAGATA  AAAAATTTTTCACGCTTACATGATTCTTTTCAGCTCCCGTATCctatttagttatttgtttag-3’ | PMA5 plasmid and upstream of PG3 downstream |
| P5 | 5’-aattaatggaaattttctaattcaacaaacgggccagttt -3’ | PMA5 plasmid and downstream of PG3 upstream |
| P6 | 5’-gggtaaaaagttatttccatTGTAATAACCTCCTCAATAGTATGAATATGTAATTAATTGTTATCCGCTCACAATTCACAATTCTTATAATAAAGAATCTCCCTTCCAATGTCA-3’ | PMA5 plasmid and downstream of PG3 upstream |
|  |  |  |
| P7 | 5-ttcaacaaacgggccagttt-3’ | PMA5 plasmid with repB removed upstream |
| P8 | 5’-ctatttagttatttgtttag-3’ | PMA5 plasmid with repB removed downstream |
| P9 | 5’-CCCGTTTTCCAGTAGAATGTATAGAAGTGTACTGCATACATACGGAAGAGGAGATGACCTATGACAAAAAAAATCCATAT-3’ | pIM1773 Gibson ligation, GtLadA and downstream of promoter region upstream |
| P10 | 5’-TTATACATTTGAAGAAATAT-3’ | pIM1773 Gibson ligation, GtLadA and downstream of promoter region downstream |
| P11 | 5’-agcggccgctgcaggcatgcTAAGTGTGCCTTTTCCTTTGCTTCAACGGTTGAACGGGCGCCCGTTTTCCAGTAGAATGT-3’ | pIM1773 Gibson ligation, GtLadA and upstream of promoter region upstream |
| P12 | 5’-gctatgaccatgattacgccTTATACATTTGAAGAAATAT-3’ | pIM1773 Gibson ligation, GtLadA and upstream of promoter region downstream |
| P13 10NNKup | 5’- ATTAATGCTNNKGAAATGAACTGCGTTGGT-3’ | Residue 10 NNK saturation mutagenesis upstream |
| P14 10NNKdown | 5’-GTTCATTTCMNNAGCATTAATGTGTATCTT -3’ | Residue 10 NNK saturation mutagenesis downstream |
| P15 12NNKup | 5’- GCTTTTGAANNKAACTGCGTTGGTCACATC -3’ | Residue 12 NNK saturation mutagenesis upstream |
| P16 12NNKdown | 5’- AACGCAGTTMNNTTCAAAAGCATTAATGTG-3’ | Residue 12 NNK saturation mutagenesis downstream |
| P17 17NNKup | 5’-TGCGTTGGTNNKATCGCGCATGGTCTGTGG-3’ | Residue 17 NNK saturation mutagenesis upstream |
| P18 17NNKdown | 5’-ATGCGCGATMNNACCAACGCAGTTCATTTC-3’ | Residue 17 NNK saturation mutagenesis downstream |
| P19 57NNKup | 5’-CTGTTCCTGNNKGACGTGGTAGGCATCTAC-3’ | Residue 57 NNK saturation mutagenesis upstream |
| P20 57NNKdown | 5’-TACCACGTCMNNCAGGAACAGTGCATCAAA-3’ | Residue 57 NNK saturation mutagenesis downstream |
| P21 58NNKup | 5’- TTCCTGGCANNKGTGGTAGGCATCTACGAC-3’ | Residue 58 NNK saturation mutagenesis upstream |
| P22 58NNKdown | 5’-GCCTACCACMNNTGCCAGGAACAGTGCATC-3’ | Residue 58 NNK saturation mutagenesis downstream |
| P23 59NNKup | 5’- CTGGCAGACNNKGTAGGCATCTACGACGTG -3’ | Residue 59 NNK saturation mutagenesis upstream |
| P24 59NNKdown | 5’- GATGCCTACMNNGTCTGCCAGGAACAGTGC-3’ | Residue 59 NNK saturation mutagenesis downstream |
| P25 79NNKup | 5’-GAGGCTGTGNNKATTCCGGTCAATGATCCG-3’ | Residue 79 NNK saturation mutagenesis upstream |
| P26 79NNKdown | 5’-GACCGGAATMNNCACAGCCTCACGAACCGC-3’ | Residue 79 NNK saturation mutagenesis downstream |
| P27 133NNKup | 5’-ATCGCGTGGNNKGTGGTGACGAGCCATTTG-3’ | Residue 133 NNK saturation mutagenesis upstream |
| P28 133NNKdown | 5’-CGTCACCACMNNCCACGCGATACGGCCTTT-3’ | Residue 133 NNK saturation mutagenesis downstream |
| P29 135NNKup | 5’- TGGAATGTGNNKACGAGCCATTTGCCAAGT-3’ | Residue 135 NNK saturation mutagenesis upstream |
| P30 135NNKdown | 5’-ATGGCTCGTMNNCACATTCCACGCGATACG -3’ | Residue 135 NNK saturation mutagenesis downstream |
| P31 227NNKup | 5’- ATTTACCAGNNKGGTATGTCGGAACGTGGT -3’ | Residue 227 NNK saturation mutagenesis upstream |
| P32 227NNKdown | 5’- CGACATACCMNNCTGGTAAATCACCGGGGT-3’ | Residue 227 NNK saturation mutagenesis downstream |
| P33 311NNKup | 5’-CACTTGGCGNNKTATGGTGGTGGTACGGGC-3’ | Residue 311 NNK saturation mutagenesis upstream |
| P34 311NNKdown | 5’- ACCACCATAMNNCGCCAAGTGACCTTCCAG-3’ | Residue 311 NNK saturation mutagenesis downstream |
| PG3 | 5’-GATACGGGAGCTGAAAAGAATCATGTAAGCGTGAAAAATTTTTTATCTTATCACTTGACATTGGAAGGGAGATTCTTTATTATAAGAATTGTGAATTGTGAGCGGATAACAATTAATTACATATTCATACTATTGAGGAGGTTATTACA-3’ | PG3 promoter |
| PuppT12 Promoter | 5’-TAAGTGTGCCTTTTCCTTTGCTTCAACGGTTGAACGGGCGCCCGTTTTCCAGTAGAATGTATAGAAGTGTACTGCATACATACGGAAGAGGAGATGACCT -3’ | GtLadA promoter |

**Table S2** Virtual saturation mutagenesis and the changes in free energy for the enzyme-ligand complex

| **Mutation** | **ΔG** | **Mutation** | **ΔG** | **Mutation** | **ΔG** | **Mutation** | **ΔG** |
| --- | --- | --- | --- | --- | --- | --- | --- |
| Ala57His | -5.0 | Val59Lys | -4.1 | Asp58His | -4.1 | Gln79His | -3.5 |
| Val135Tyr | -3.5 | Asn133His | -3.3 | Ala227Lys | -2.8 | Tyr63His | -2.8 |
| Phe10Lys | -2.4 | His17Cys | -2.3 | His311Cys | -2.3 |  |  |

**ΔG unit. kJ/mol**

**Table S3** Alkane degradation rate (ADR) for *G. thermodenitrificans* GZ156, and *G. stearothermophilus* GZ178

|  | **Alkane degradation rate (ADR)** | | |
| --- | --- | --- | --- |
|  | ***G. t* GZ156** | ***G. s* GZ178** | ***G. s* GZ178 (pIM*PpladA2mu*)** |
| dodecane | (91.8 ± 0.8)% | N.D | N.D |
| tetradecane | (87.5± 0.9)% | N.D | N.D |
| tetramethyldodecane | (79.6± 0.7)% | N.D | N.D |
| pentadecane | (68.8± 0.6)% | N.D | (9.7± 0.4)% |
| hexadecane | (40.7± 0.2)% | N.D | (10.0± 0.2)% |
| trimethylpentadecane | (26.5± 0.1)% | N.D | (7.4± 0.2)% |
| dimethyl heptadecane | (8.4± 0.1)% | N.D | (6.1± 0.1)% |
| heptadecane | (6.7± 0.1)% | N.D | (9.4± 0.5)% |
| octadecane | N.D | (3.9± 0.1)% | (11.7± 0.3)% |
| nonadecane and eicosane | N.D | (10.2± 0.2)% | (16.3± 0.3)% |
| eicosane | N.D | (11.7± 0.3)% | (14.1± 0.2)% |
| docosane | N.D | (13.0± 0.2)% | (18.2± 0.4)% |
| tricosane | N.D | (10.9± 0.1)% | (14.1± 0.2)% |
| tetracosane | N.D | (13.8± 0.2)% | (17.1± 1.3)% |
| hexacosane | N.D | (6.9± 0.1)% | (10.5± 0.6)% |
| heptacosane | N.D | (42.1± 1.2)% | (52.4± 2.7)% |
| octacosane | N.D | (44.3± 0.9)% | (51.4± 1.6)% |

ADR =$\frac{P2- P1}{P2}\times100\%$, where P2 is the total peak area of the alkane control on gas chromatography (GC), and P1 represents the total peak area of the alkane-degrading strain on GC.

The degradation experiment was performed in triplicate.

N.D, not detected
