# Supplementary figures and images for: Development of a microbial dewaxing agent using three spore forming bacteria
Source: Bioresour Bioprocess. 2024 Aug 8;11(1):80. doi: 10.1186/s40643-024-00795-z (PMC11310373; doi:10.1186/s40643-024-00795-z)

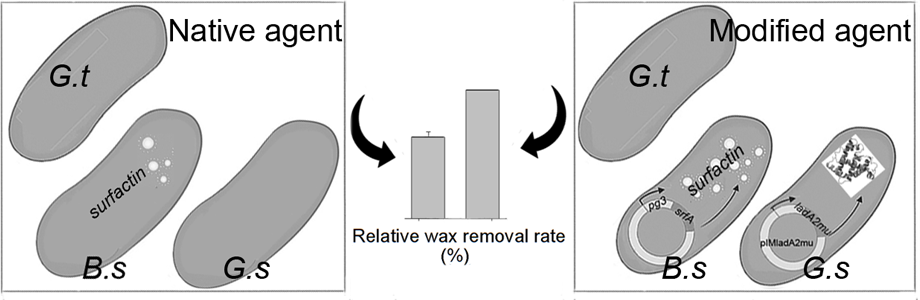

Supplement: Supplementary file 4 — Supplementary Material 4 [file 40643_2024_795_MOESM4_ESM.png]
