# Supplementary material for: Development of a microbial dewaxing agent using three spore forming bacteria
Source: Bioresour Bioprocess. 2024 Aug 8;11(1):80. doi: 10.1186/s40643-024-00795-z (PMC11310373; doi:10.1186/s40643-024-00795-z)
Supplement: Supplementary file 3 — Supplementary Material 3 [file 40643_2024_795_MOESM3_ESM.docx]

Reviewer #1: For the note " I prefer another keyword than microbial wax removal agent, not included in the title", I mean that the keywords should be different from the words mentioned in the title. So, you can let "microbial wax removal agent" in the keyword section and use "microbial dewaxing agent" in the title.

Respond:

We revised the keyword list as reviewer suggested.
